# *Mycobacterium tuberculosis* DevR/DosR Dormancy Regulator Activation Mechanism: Dispensability of Phosphorylation, Cooperativity and Essentiality of α10 Helix

**DOI:** 10.1371/journal.pone.0160723

**Published:** 2016-08-04

**Authors:** Saurabh Sharma, Jaya Sivaswami Tyagi

**Affiliations:** Department of Biotechnology, All India Institute of Medical Sciences, Ansari Nagar, New Delhi, India; Infectious Disease Research Institute, UNITED STATES

## Abstract

DevR/DosR is a well-characterized regulator in *Mycobacterium tuberculosis* which is implicated in various processes ranging from dormancy/persistence to drug tolerance. DevR induces the expression of an ~48-gene dormancy regulon in response to gaseous stresses, including hypoxia. Strains of the Beijing lineage constitutively express this regulon, which may confer upon them a significant advantage, since they would be ‘pre-adapted’ to the environmental stresses that predominate during infection. Aerobic DevR regulon expression in laboratory-manipulated overexpression strains is also reported. In both instances, the need for an inducing signal is bypassed. While a phosphorylation-mediated conformational change in DevR was proposed as the activation mechanism under hypoxia, the mechanism underlying constitutive expression is not understood. Because DevR is implicated in bacterial dormancy/persistence and is a promising drug target, it is relevant to resolve the mechanistic puzzle of hypoxic activation on one hand and constitutive expression under ‘non-inducing’ conditions on the other. Here, an overexpression strategy was employed to elucidate the DevR activation mechanism. Using a panel of kinase and transcription factor mutants, we establish that DevR, upon overexpression, circumvents DevS/DosT sensor kinase-mediated or small molecule phosphodonor-dependent activation, and also cooperativity-mediated effects, which are key aspects of hypoxic activation mechanism. However, overexpression failed to rescue the defect of C-terminal-truncated DevR lacking the α10 helix, establishing the α10 helix as an indispensable component of DevR activation mechanism. We propose that aerobic overexpression of DevR likely increases the concentration of α10 helix-mediated active dimer species to above the threshold level, as during hypoxia, and enables regulon expression. This advance in the understanding of DevR activation mechanism clarifies a long standing question as to the mechanism of DevR overexpression-mediated induction of the regulon in the absence of the normal environmental cue and establishes the α10 helix as an universal and pivotal targeting interface for DevR inhibitor development.

## Introduction

Two component systems (TCS) permit bacteria to sense and adapt to diverse environmental stresses [1]. The DevR-DevS TCS (also called DosR-DosS) is one of the best characterized TCS of *Mycobacterium tuberculosis* (Mtb). It is induced by multiple gaseous stresses, including hypoxia [2] and also by vitamin C, which leads to hypoxia [3]. One or more of these inducing conditions are thought to prevail inside granulomas wherein Mtb can survive indefinitely, sometimes for decades, in a dormant state. DevR is believed to be one of the key regulators that mediate Mtb adaptation to a dormant state during infection. This is supported by findings in a macaque model of tuberculosis wherein long-term persistence was compromised upon infection with a *devR* knockout (RKO) strain of Mtb [4]. Under inducing conditions, DevR is activated by transfer of the phosphosignal from either DevS or DosT or both sensor kinases [5–7], which leads to the induction of ~48 genes, comprising the DevR regulon [8]. This regulon is not induced in a *devS* and *dosT* Mtb mutant that expresses *devR* (DKO), establishing the critical role of phosphosignaling in induction [3]. DevR-independent transcription of the *Rv3134c-devRS* operon maintains the aerobic basal level of DevR [9, 10], and under inducing conditions, positive autoregulation results in a phosphorylation-dependent increase in *devR* transcription [11] and a corresponding ~5-fold increase in DevR protein level [10].

Based on the crystal structure of full length DevR, it was proposed that DevR must undergo significant phosphorylation-dependent conformational changes under inducing conditions to bind to target DNA [12]. It is established that cooperative binding of phosphorylated DevR to target promoters is essential for regulon activation [13, 14]. Mtb strains of the Beijing lineage express *devR* at ~50 fold higher level under aerobic/non-inducing conditions in comparison to other strains [15]. For a number of regulators, the natural phosphorylation mechanism can be bypassed by artificially overexpressing the response regulator; e.g. PhoP of *Salmonella enterica* [16], UhpA in *E*. *coli* [17] and DevR in Mtb. The overexpression of DevR in H37Rv Δ*devR* [18] and in H37Rv [19] led to aerobic expression of the regulon. While the physiological relevance of aerobic expression can be interrogated, it also leaves open the question of DevR activation mechanism and the role of phosphorylation and cooperativity in regulon induction under aerobic conditions. Moreover, the possibility remains of phosphorylated regulator species being generated by crosstalk from non-cognate sensors or small molecule phosphodonors. Thus, the mechanism of DevR regulon induction in aerobic conditions under overexpression studies remains a puzzle and very important to decipher, in the context of targeting DevR, especially in Beijing strains, as a novel dormancy drug target.

In the light of these observations, the present study aimed to elucidate the mechanism underlying aerobic induction of the DevR regulon, that is under ‘non-inducing’ conditions. Wild type and mutant forms of DevR, defective in key activation functions, including phosphorylation, cooperative binding and dimerization, were examined for their role in aerobic activation under DevR overexpression conditions in Mtb. This study has provided important insights into the mechanism of activation under physiological conditions (hypoxia) as well as in the overexpression scenario which will facilitate the development of novel inhibitors of DevR.

## Materials and Methods

### Culture conditions

Stocks of Mtb H37Rv-derived strains stored at– 80°C were grown as primary and secondary cultures in Middlebrook 7H9 medium containing 0.05% Tyloxapol and ADS supplement (0.5% Albumin, 0.75% Dextrose, 0.085% NaCl), with shaking at 37°C (10 ml in 50 mL tubes). Antibiotics were used at the following concentrations: hygromycin (Hyg), 50 μg/mL for Mtb and 200 μg/mL for *E*. *coli*; kanamycin (Kan), 25 μg/mL for Mtb and 50 μg/mL for *E*. *coli*. Strictly aerobic conditions were maintained by growing cultures with vigorous aeration at 220 rpm to an OD_590_ of 0.2–0.3, by rapidly harvesting the cultures by centrifugation for 10 minutes at 4°C in pre-chilled rotors, immediately resuspending the pellet in TRI solution (containing guanidine thiocyanate and phenol) and storing at -80°C. For the hypoxia set-up, standing cultures were incubated at 37°C for 5 days as described [20].

### Construction of Mtb strains

Plasmids that overexpress DevR-Myc (**S1 Table**) were generated by replacement of *Rv3134c-devRS* operon promoter in pKK P_Operon_DevR-Myc with *hsp60*, *msp12* or *rrn* promoter sequences generated by PCR (**S2 Table)**. pTEC15 used as template for amplification of *msp12* promoter [21] was a gift from Lalita Ramakrishnan (Addgene plasmid # 30174). Site-directed mutagenesis was performed as described [20] to generate all mutant *devR* expressing plasmids, except pSS P_msp12_DevR D54V-Myc which was constructed by replacing the operon promoter with *msp12* promoter in pKK P_Operon_DevR D54V-Myc (**S1 Table**). pSS P_msp12_DevR_*Δ*α10_ was generated by replacing the operon promoter with *msp12* promoter in pAV P_Operon_DevR_Δα10_. All the recombinant Mtb strains were constructed by electroporation of integration-proficient recombinant plasmids (**S1 Table**) in Mtb DKO and RKO strains (**Table 1)**. Mtb DKO is a double sensor kinase knockout strain of H37Rv generated by disrupting *devS* by gene replacement with a kanamycin resistance determinant, and *dosT* by gene replacement with a mutated copy of *dosT* that contained premature stop codons [5, 22]. This strain expresses DevR from its native promoter [23]. Mtb RKO is a *devR* knockout strain of H37Rv generated by deleting a 447-bp region of *devR* [24].

**Table 1:** Mtb strains used in the study.

Strain	Mtb background	Complementing plasmid	DevR over-expression	Expression	Source
RKO	H37Rv Δ*devR*	-	-	-	[24]
DKO	H37Rv Δ*dosS*Δ*dosT*	-	-	Endo* WT DevR	[5]
DKO-P_hsp60_DevR	DKO	pSS P_hsp60_DevR-Myc	Yes	Endo WT DevR + DevR-Myc	This study
DKO-P_msp12_DevR	DKO	pSS P_msp12_DevR-Myc	Yes	Endo WT DevR + DevR-Myc	This study
DKO-P_rrn_DevR	DKO	pSS P_rrn_DevR-Myc	Yes	Endo WT DevR + DevR-Myc	This study
RKO-P_Operon_DevR	RKO	pKK P_Operon_DevR-Myc	No	Endo WT DevR + DevR-Myc	Dr. Kohinoor Kaur, (unpublished results)
RKO-P_msp12_DevR	RKO	pSS P_msp12_DevR-Myc	Yes	WT DevR-Myc	This study
RKO-P_msp12_DevR D54E	RKO	pSS P_msp12_DevR D54E-Myc	Yes	DevR D54E-Myc	This study
RKO-P_msp12_DevR D54V	RKO	pSS P_msp12_DevR D54V-Myc	Yes	DevR D54V-Myc	This study
RKO-P_msp12_DevR T82A	RKO	pSS P_msp12_DevR T82A-Myc	Yes	DevR T82A-Myc	This study
RKO-P_msp12_DevR_*Δ*α10_	RKO	pSS P_msp12_DevR_*Δ*α10_	Yes	DevR_*Δ*α10_	This study

* Endo, endogenous

### RNA isolation, RT-qPCR and Immunoblotting

Mtb cultures grown as mentioned above were processed for RNA isolation as described previously [11]. For RT-qPCR, cDNA was synthesized using 200 ng of total RNA, 50 units of Multiscribe reverse transcriptase and random hexamer primers as per the manufacturer’s instructions (Applied Biosystems, USA). Two microlitres of cDNA was subjected to qPCR using gene specific primers (**S3 Table**) and iQ SyBr Green Supermix (Bio-Rad, USA) in a 25 μl reaction mixture in CFX96 Real-Time PCR-detection system (Bio-Rad, USA). ΔΔC_t_ method was employed to normalize test samples with 16S rRNA transcripts and to calculate relative fold change in expression with respect to DKO or RKO-P_Operon_DevR strains. Experiments were performed using two biological and technical replicates each. For data analysis, 2 technical replicates were averaged to generate one value (a biological replicate value). Two biological replicate values for each strain were then used to plot graphs having mean and standard deviation.

For lysate preparation, the aerobic and hypoxic Mtb cultures were harvested and stored at -80°C. Lysates were prepared from two cultures as described previously [25]. DevR, SigA and HspX proteins were detected in the lysates (~15 μg) by Western blotting using anti-Myc (Sigma Aldrich), anti-DevR, anti-SigA and anti-HspX antibodies (generated in house in Central Animal Facility, AIIMS) as described earlier [9].

## Results

### Comparative expression of DevR from various mycobacterial promoters in Mtb DKO

**Fig 1:**
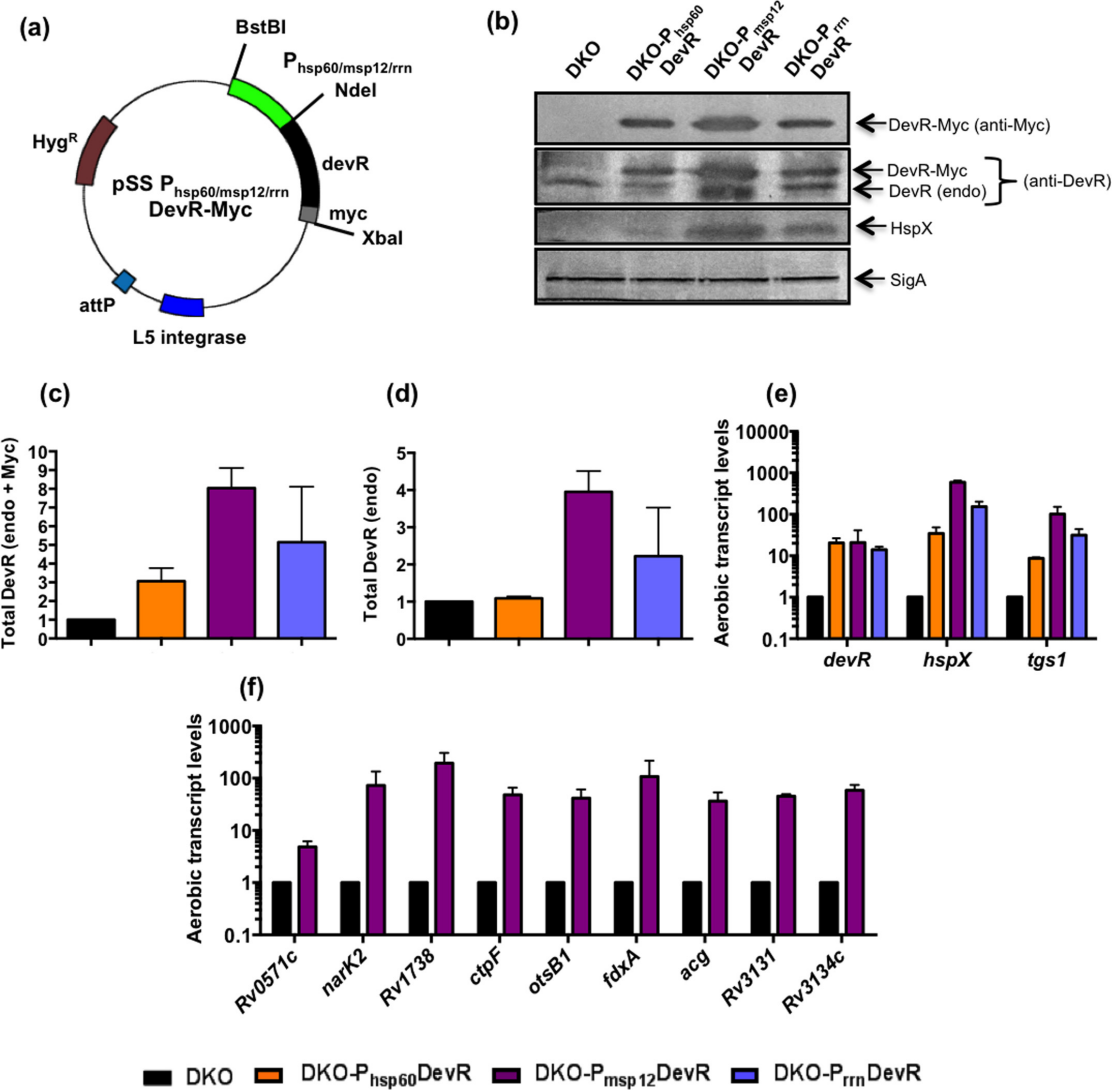
Overexpression of DevR in Mtb DKO. **(a)** Vector map of integrating plasmid overexpressing DevR. **(b)** Western blotting of lysates from aerobic Mtb cultures. A representative blot is shown. **(c)** Total DevR levels (DevR + DevR-Myc) in DKO-complemented strains. **(d)** DevR (endo) level in DKO-complemented strains. **(e)** RT-qPCR analysis of select DevR regulon genes in DKO-complemented strains. **(f)** RT-qPCR analysis of additional DevR regulon genes in DKO-P_msp12_DevR. Data is Mean ± SD of 2 biological replicates. SigA was used as a loading control in (b). In panels (c) to (f), protein and transcript levels are shown relative to that in DKO (considered as 1).

Three mycobacterial promoters, namely Mtb *hsp60* (DKO-P_hsp60_DevR), *M*. *marinum msp12* (DKO-P_msp12_DevR) and Mtb *rrn* (DKO-P_rrn_DevR) were cloned upstream of wild-type *devR* sequence (*myc* tagged, **Fig 1A**). These constructs were electroporated in Mtb DKO strain and the resulting strains (**Table 1**) were compared for the expression of DevR by western blotting using anti-Myc and anti-DevR antibodies. Relative promoter strengths were assessed by comparing DevR levels using anti-Myc antibody. The *msp12* promoter (DKO-P_msp12_DevR) supported the maximal expression of Myc-tagged DevR (**Fig 1B**). Aerobic cultures of DKO-P_hsp60_DevR, P_msp12_DevR and P_rrn_DevR expressed DevR at ~3- to 8-fold higher level compared to DKO (**Fig 1C**). Two species of DevR were detected with anti-DevR antibody; native DevR expressed from the endogenous gene (DevR endo) and ectopically expressed DevR-Myc (Myc-tagged). DKO-P_msp12_DevR supported maximum total DevR expression (~8-fold relative to DKO, **Fig 1C**). The level of DevR endo was also highest in DKO- P_msp12_DevR (**Fig 1D**) and this is attributed to positive autoregulation of the native copy of *devR* as described earlier [11]. These findings provided the first line of evidence that high level expression of DevR auto-induces *devR* under aerobic conditions (considered to be ‘non-inducing’) in these strains. The expression of dormancy antigen HspX, another member of the regulon, was also highest in DKO-P_msp12_DevR and not detectable in DKO, which expresses DevR only at basal levels. An ~21-fold overexpression of *devR* in DKO-P_msp12_DevR was quite comparable to ~50-fold overexpression reported in Beijing strains [15]. The aerobic induction of select regulon genes was also maximal in DKO-P_msp12_DevR (**Fig 1E**). DKO-P_msp12_DevR was investigated further for expression of 9 additional DevR regulon genes, each of which was notably induced compared to the background DKO strain **(Fig 1F)**. These findings established that DevR induces the aerobic expression of regulon genes under conditions of overexpression and in the absence of its cognate sensor kinases, DevS and DosT. This observation raised the question of how was DevR activated and the regulon induced under aerobic conditions.

### Overexpression of WT and phosphorylation defective mutant DevR proteins in Mtb RKO

In the absence of cognate sensor kinases in the DKO complemented strains, the observation that DevR overexpression causes aerobic expression of the regulon can, in principle, be attributed to DevR being phosphorylated by small molecule phosphodonors, like acetyl phosphate (AcP). Indeed, there is growing evidence in the literature, of response regulators such as RcsB, NtrC, OmpR, CpxR, VanR, Rrp2 and VicR, being phosphorylated by AcP [26]. Therefore, to investigate this possibility, two Mtb strains were constructed which overexpress WT or phosphorylation defective mutant forms of DevR (DevR D54V and D54E). These substitution mutants were earlier shown to be phosphorylation defective [5, 6, 8].

**Fig 2:**
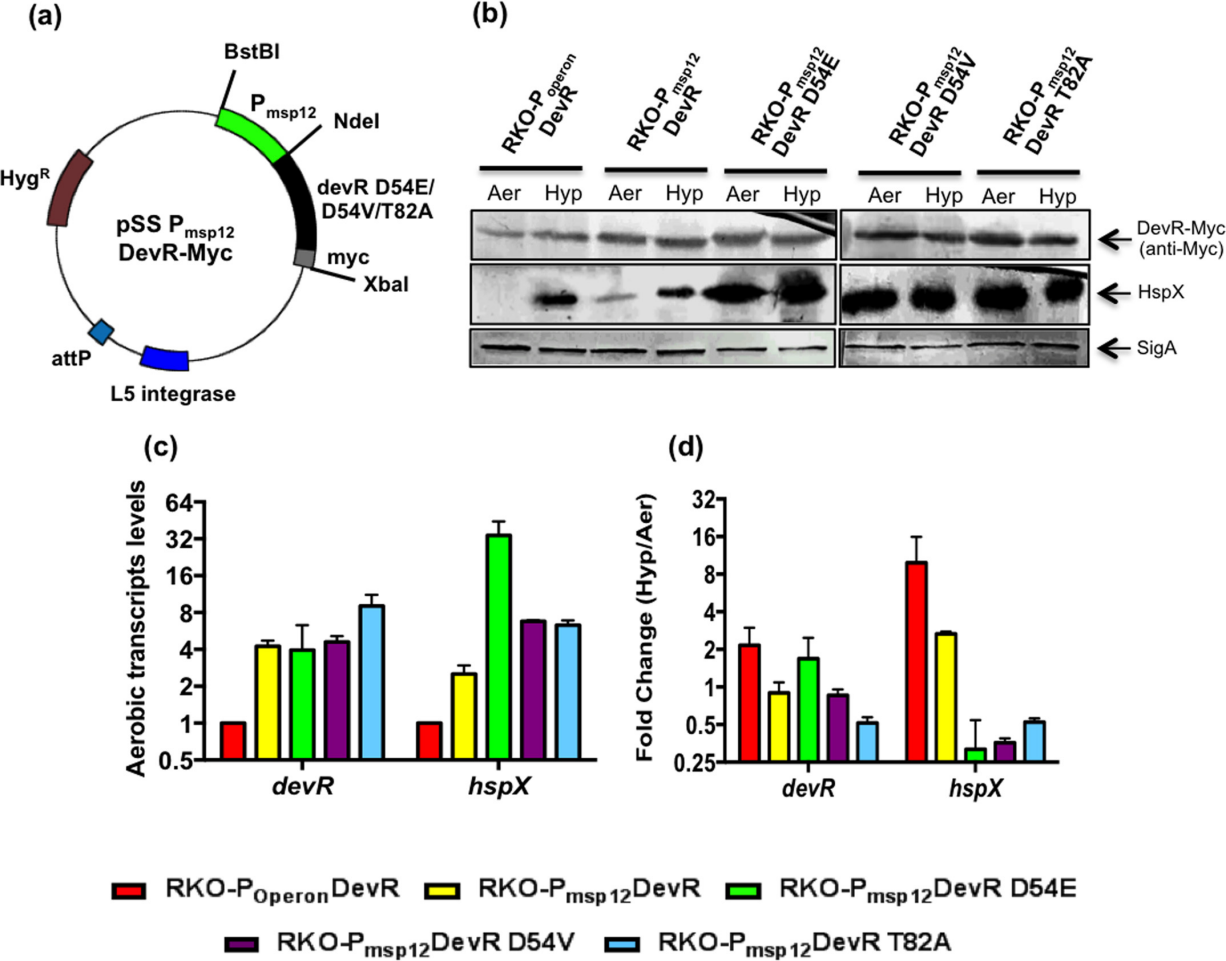
Overexpression of WT/mutant DevR in Mtb RKO. **(a)** Vector map of integrating plasmid overexpressing WT/mutant DevR proteins. **(b)** Mtb RKO- P_msp12_DevR, RKO-P_msp12_DevR D54E, RKO-P_msp12_DevR D54V and RKO-P_msp12_DevR T82A strains overexpress DevR variants from *msp12* promoter. RKO-P_Operon_DevR strain expresses DevR from its native promoter. Western blotting of aerobic (Aer) and 5 day hypoxic (Hyp) cultures lysates. A representative blot is shown. **(c)** RT-qPCR analysis of regulon genes in aerobic cultures. **(d)** RT-qPCR analysis of DevR regulon genes under hypoxia. RT-qPCR data is Mean ± SD of 2 biological replicates. SigA was used as a loading control in (b).

Mtb complemented strains (RKO-P_msp12_DevR, P_msp12_DevR D54E and P_msp12_DevR D54V) were constructed in RKO background, which expresses DevS and DosT kinases and is deleted of *devR* gene (**Table 1**). RKO-P_msp12_DevR, which expresses WT DevR-Myc from *msp12* promoter, supported the aerobic expression of *hspX* transcripts and HspX protein (**Fig 2B and 2C**). The role of phosphorylation in DevR activation mechanism was interrogated in RKO-P_msp12_DevR D54E and P_msp12_DevR D54V strains overexpressing phosphorylation-defective variants, DevR D54E and DevR D54V, respectively (**Table 1**). The *hspX* gene was induced at the RNA and protein levels in both these strains (**Fig 2B and 2C**). Notably, DevR D54E (RKO-P_msp12_DevR D54E) supported higher expression of *hspX* transcripts than either WT DevR (RKO-P_msp12_DevR) or DevR D54V mutant (RKO-P_msp12_DevR D54V). These findings established unequivocally that (1) DevR, when overexpressed, induces aerobic DevR regulon expression, (2) the extent of activation depends upon the level of DevR expression, and (3) under overexpression conditions, the phosphorylation/ inducing signal is dispensable.

### Overexpression of cooperativity-defective mutant DevR T82A overrides defect in regulon induction

Cooperative binding of DevR to tandemly arranged sites on DNA was shown to be essential for the activation of regulon genes [27]. It was also shown that DevR T82A mutant protein, when expressed from its native promoter, abrogated regulon expression due to a defect in cooperative binding [14]. Next, to assess whether, like phosphorylation, T82-mediated cooperative interactions could be bypassed via protein overexpression, and enable aerobic regulon expression, DevR T82A was overexpressed in RKO-P_msp12_DevR T82A. Notably, DevR T82A mutant protein also supported aerobic expression of HspX at transcript and protein levels (**Fig 2**), establishing that overexpression also compensated for defects in T82-mediated cooperativity.

### α10 helix of DevR is indispensable for DevR activation

**Fig 3:**
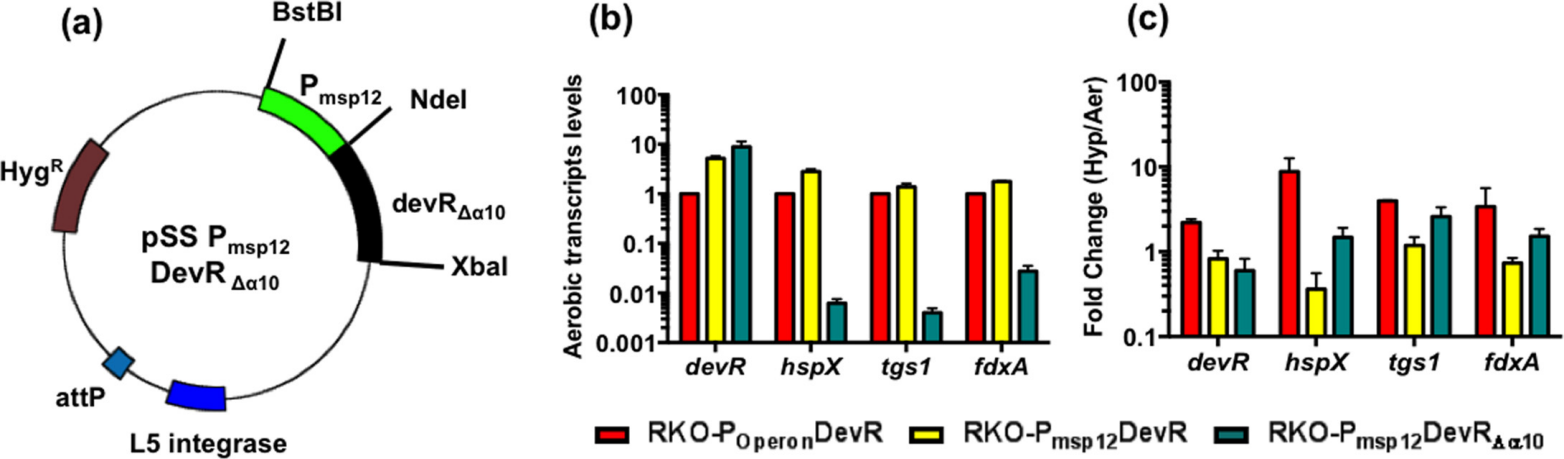
Overexpression of DevR_Δα10_ in Mtb RKO. **(a)** Vector map of integrating plasmid employed to overexpress DevR_α10_ protein. **(b)** RT-qPCR analysis of aerobic DevR regulon genes expression. WT/mutant DevR expression in RKO-P_msp12_DevR /RKO-P_msp12_DevR_α10_ strains is supported by *msp12* promoter and WT DevR in RKO-P_Operon_DevR by its native promoter. **(c)** RT-qPCR analysis of DevR regulon genes under hypoxia. RT-qPCR data is Mean ± SD of 2 biological replicates.

It was proposed from modeling crystal structure data that extensive structural rearrangement would occur in DevR protein upon phosphorylation and the α10 helix may be necessary to form the active DevR dimer species [12]. Recently, the α10 helix was experimentally established to be essential for regulon activation under hypoxia [28]. The role of this helix in aerobic DevR activation mechanism under overexpression conditions was examined next in RKO-P_msp12_DevR_*Δ*α10_, which expresses a C-terminally truncated DevR protein lacking this helix (DevR_Δα10_ contains 1–193 amino acid residues, **Fig 3A**). In contrast to other complemented strains, RKO- P_msp12_DevR_*Δ*α10_ was completely defective in regulon genes expression under aerobic as well as hypoxic conditions (**Fig 3B and 3C).** Interestingly, even though RKO-P_msp12_DevR_*Δ*α10_ expressed *devR* at ~9-fold higher levels compared to RKO-P_Operon_DevR, the level of regulon genes expression in the former strain under aerobic condition was only ~ 1/100^th^ compared to the latter. Higher levels of regulon transcripts in the latter strain could be due to very minor signaling through DevS and DosT even under strictly aerobic conditions, while the expression was defective in RKO-P_msp12_DevR_*Δ*α10_ because of the presence of truncated DevR protein. Thus the activation defect in DevR_Δα10_ is not rescued by overexpression of this truncated protein.

### DevR overexpression under hypoxia

Various complemented strains in Mtb RKO background were also analyzed to determine the effect of WT and mutant protein overexpression on hypoxic induction. Not surprisingly, only P_Operon_DevR and P_msp12_DevR exhibited regulon genes induction. Both these strains express WT DevR and can therefore be activated by phosphorylation under hypoxia (**Fig 2**). In contrast, P_msp12_DevR D54E, P_msp12_DevR D54V and P_msp12_DevR T82A strains, which constitutively overexpress defective DevR proteins, did not support a further increase in regulon genes expression over the aerobically ‘pre-induced’ level (**Fig 2C**).

## Discussion

The first key finding of this study is that DevR overexpression overrides the physiological control of phosphorylation that is reportedly essential for its activation [12, 27]. There are examples of regulators whose overexpression bypasses sensor kinase-dependent activation, including *Salmonella enterica* PhoP [16] and *E*. *coli* UhpA [17]. *Pseudomonas aeruginosa* PilR overexpression rescued pilin expression; however, cross-phosphorylation by a small molecule phosphodonor/ another histidine kinase could not be ruled out [29]. Likewise, the overexpression of *Rhizobium meliloti* FixJ activated target gene expression, although again the possibility of FixJ being cross-phosphorylated was not excluded [30]. There are two reports of manipulated DevR overexpression in Mtb, but these strains expressed DevS and DosT kinases [18, 19]. However, the mechanism underlying regulator activation was not addressed. Thus an activation mechanism to explain aerobic expression of the regulon that was also consistent with hypoxic activation was missing. Here we demonstrate that DevR, at high concentration, bypasses sensor kinase/ small molecule phosphodonor-dependent activation. To the best of our knowledge, this is the first study to assess the role of phosphorylation and cooperative interactions in the activation mechanism of any overexpressed regulator and to decipher the activation mechanism under ‘non-inducing’ conditions.

The robust expression of regulon genes observed in the presence of overexpressed DevR D54E is noteworthy. This is in contrast to a previous report [8], wherein this mutant protein did not mediate induction of *hspX* under aerobic and hypoxic conditions. The discrepant result may be attributed to differences in DevR level and strain background; constitutive overexpression in Mtb (present study) vs. native promoter-driven expression in BCG [8]. This mutation in other systems too was shown to bypass phosphorylation, such as NtrC [31], AlgR [32] and ComE [33], wherein it was suggested that a glutamic acid substitution acts as a phosphomimic.

The ability of protein overexpression to override phosphorylation/ cooperativity defects in DevR raises two important questions pertaining to (1) the protein conformation of active DevR under overexpression and ‘non-inducing’ aerobic conditions, and (2) the role of protein concentration (overexpression) in adopting the active conformation. From the crystal structure it was proposed that phosphorylation-driven conformational changes could occur in hypoxia to generate the active species via α10 helix-mediated dimerization [12]. Recently, the critical role of the α10 helix was experimentally established under hypoxia; genetic deletion of the α10 helix (DevR_*Δ*α10_) abrogated regulon induction under hypoxia [28]. Here, we show that overexpression of this truncated protein failed to rescue this defect in aerobic cultures as well (**Fig 3**). The activation defect of DevR_*Δ*α10_ protein can be attributed to (1) absence of α10 helix-mediated dimer, and /or (2) defect in folding of the remaining protein. The latter possibility is excluded; both DevR_*Δ*α10_ and WT proteins form dimers in the unphosphorylated state [28], implying that the interfaces are properly folded. However, while WT protein formed dimers post-phosphorylation, phosphorylated α10 helix-deleted protein did not [28]. Taking all the results together, it is concluded that it is the absence of α10 helix-mediated active dimer species, and not protein folding defect, which defines the inability of DevR_*Δ*α10_ overexpressing strain to support regulon genes expression. A critical question still remains: how is the α10 helix-mediated active dimer species formed in aerobic conditions without a phosphostimulus? A role for protein concentration appears likely under overexpression conditions. It is believed that response regulators including DevR exist in an equilibrium of inactive/ active conformations, and phosphorylation drives the equilibrium towards the latter under inducing conditions [12]. The α10 helix and tail were proposed to play a key role in DevR rearrangement to form the active species [12]. However, this rearrangement is not feasible in DevR_*Δ*α10_ protein. Based on the findings of the present study, we propose that the active dimer concentration is maintained below the threshold level in strains that express DevR at endogenous levels. What protein overexpression does is to increase the concentration of the active conformer above the inducing threshold under aerobic conditions, resulting in regulon genes expression. In the former, DevR is activated through phosphorylation mechanism, while in the latter, the increase in concentration of α10 helix-mediated dimer compensates for absence of the inducing stimulus. A higher degree of regulon induction was observed in D54E and D54V compared to WT DevR-expressing strain. This finding can be explained by the loss of interaction between (E/V)54 and Q199 residues as predicted from the crystal structure [12], and may favour the formation of transcription proficient α10 helix-mediated dimers. In addition, it is plausible that the longer, negatively charged side chain length in E residue, vs. V residue, contributes to greater repulsion between the N and C terminal domains favouring more formation of active dimers and hence greater induction. Thus, the second key finding of this study is that α10 helix-mediated dimer formation is absolutely essential in DevR activation mechanism under overexpression as well as natural inducing conditions (such as hypoxia). These advances in our understanding of DevR activation mechanism have provided a mechanistic insight into DevR regulon expression in clinically relevant Beijing strains of Mtb, wherein regulon genes are constitutively overexpressed and are proposed to confer a significant advantage during intracellular survival [15].

DevR-DevS is a well-characterized signal transduction pathway and DevR is a promising drug target in view of its importance for bacterial persistence. This signaling pathway has been effectively intercepted at various steps; sensor kinase autophosphorylation [34], DNA binding [35], and interaction with RNA polymerase [36], and has led to a failure in adaptation to hypoxia, a key stress signal within granulomas [10, 20]. The present study highlights the overarching importance of α10 helix-mediated dimerization as an interface for blocking DevR function under both inducing (hypoxic) conditions as well as in ‘non-inducing’ (aerobic) conditions, including in Mtb clinical strains such as Beijing strains that constitutively overexpress *devR*. This finding along with the demonstrated role of phosphorylation-induced dimerization in DNA binding of FixJ [37] and StyR [38] highlights the relevance of dimerization as an essential step in the process of gene activation. In conclusion, this universal activation mechanism for DevR involving α10 helix will enable the design of new and novel anti-tubercular drugs.

## Supporting Information

S1 Table Plasmids used in this study. (PDF)

S2 Table Oligonucleotide primers used for PCR amplification. (PDF)

S3 Table Oligonucleotide primers used for qPCR. (PDF)
