# Supplementary material for: Mycobacterium tuberculosis DevR/DosR Dormancy Regulator Activation Mechanism: Dispensability of Phosphorylation, Cooperativity and Essentiality of α10 Helix
Source: PLoS One. 2016 Aug 4;11(8):e0160723. doi: 10.1371/journal.pone.0160723 (PMC4973870; doi:10.1371/journal.pone.0160723)
Supplement: S1 Table — (PDF) [file pone.0160723.s001.pdf]

S1 Table. Plasmids used in this study

Plasmid	Features	Source / Reference
pJFR19	*E. coli-Mycobacterium* integrating shuttle plasmid with 3-kb acetamidase promoter, Hyg^r (parent vector used for constructing various plasmids)	[1]
pMSP12::Wasabi (pTEC15)	*E. coli-Mycobacterium* shuttle plasmid (pFPV27 background) having *msp12* promoter (from *M. marinum*) driven Wasabi coding sequences, Hyg^R	[2]
pKK P_{Operon}DevR-Myc	pJFR19* containing wt *devR-myc* expressed from Mtb *Rv3134c-devRS* operon promoter (-608 to +998), Hyg^R, expresses wt DevR	Dr. Kohinoor Kaur (Unpublished results)
pKK P_{Operon}DevR D54V-Myc	pJFR19 containing *devR D54V-myc* expressed from operon promoter (as above), Hyg^R, expresses phosphorylation defective DevR	Dr. Kohinoor Kaur (Unpublished results)
pUS P_{hsp60}*devR*$_C$	pJFR19 containing *devR*$_C$ (141-217 amino acids) expressed from *hsp60* promoter, Hyg^R, expresses DevR C-terminal domain	[3]
pSS P_{rrn}DevR-Myc	pJFR19 containing *devR-myc* expressed from *rrn* promoter, Hyg^R, expresses wt DevR	This study
pSS P_{hsp60}DevR-Myc	pJFR19 containing *devR-myc* sequence expressed from *hsp60* promoter, Hyg^R, expresses wt DevR	This study
pSS P_{msp12}DevR-Myc	pJFR19 containing *devR-myc* sequence expressed from *msp12* promoter, Hyg^R, expresses wt DevR	This study
pSS P_{msp12}DevR D54E-Myc	pJFR19 containing *devR D54E-myc* expressed from *msp12* promoter, Hyg^R, expresses phosphorylation defective DevR	This study
pSS P_{msp12}DevR D54V-Myc	pJFR19 containing *devR D54V-myc* sequence expressed from *msp12* promoter, Hyg^R, expresses phosphorylation defective DevR	This study
pSS P_{msp12}DevR T82A-Myc	pJFR19 containing *devR T82A-myc* sequence expressed from *msp12* promoter, Hyg^R, expresses cooperativity defective DevR	This study
pAV P_{Operon}DevR$_{\Delta\alpha10}$	pJFR19 containing *devR*Δα10 (DevR 1-193 amino acids), expressed from operon promoter, Hyg^R, expresses DevR deleted of α10 helix	[4]
pSS P_{msp12}DevR$_{\Delta\alpha10}$	pJFR19 containing *devR*Δα10 (DevR 1-193 amino acids), expressed from *msp12* promoter, Hyg^R, expresses DevR deleted of α10 helix and tail (194-217 amino acids)	This study

*In pJFR19 constructs expressing DevR wt/mutant proteins, the gene sequences were cloned between NdeI and XbaI sites and various promoters (*rrn*, *msp12,* etc.) between BstBI and NdeI sites.

References

1. Chauhan A, Madiraju MV, Fol M, Lofton H, Maloney E, Reynolds R, et al. Mycobacterium tuberculosis cells growing in macrophages are filamentous and deficient in FtsZ rings. J Bacteriol. 2006;188(5):1856-65. doi: 10.1128/JB.188.5.1856-1865.2006. PubMed PMID: 16484196; PubMed Central PMCID: PMC1426569.
2. Chan K, Knaak T, Satkamp L, Humbert O, Falkow S, Ramakrishnan L. Complex pattern of Mycobacterium marinum gene expression during long-term granulomatous infection. Proc Natl Acad Sci U S A. 2002;99(6):3920-5. doi: 10.1073/pnas.002024599. PubMed PMID: 11891270; PubMed Central PMCID: PMC122624.
3. Gautam US, Chauhan S, Tyagi JS. Determinants outside the DevR C-terminal domain are essential for cooperativity and robust activation of dormancy genes in Mycobacterium tuberculosis. PLoS One. 2011;6(1):e16500. doi: 10.1371/journal.pone.0016500. PubMed PMID: 21304599; PubMed Central PMCID: PMC3029386.
4. Vashist A, Prithvi Raj D, Gupta UD, Bhat R, Tyagi JS. The alpha10 helix of DevR, the Mycobacterium tuberculosis dormancy response regulator, regulates its DNA binding and activity. The FEBS journal. 2016;283(7):1286-99. doi: 10.1111/febs.13664. PubMed PMID: 26799615.
