# Supplementary material for: Mycobacterium tuberculosis DevR/DosR Dormancy Regulator Activation Mechanism: Dispensability of Phosphorylation, Cooperativity and Essentiality of α10 Helix
Source: PLoS One. 2016 Aug 4;11(8):e0160723. doi: 10.1371/journal.pone.0160723 (PMC4973870; doi:10.1371/journal.pone.0160723)
Supplement: S2 Table — (PDF) [file pone.0160723.s002.pdf]

S2 Table. Oligonucleotide primers used for PCR amplification

Primer Name	Sequence 5' → 3'	Template	Name of Plasmid generated
hsp60 BstBI F	CCGTTCGAAGGTGACCACAACGACGCGCCCGC	Mtb H37Rv DNA	pSS P_{hsp60}DevR-Myc
hsp60 NdeI R	CCGCATATGTGCGAAGTGATTCCTCCGGATCG		
msp12 BstBI F	GCCGCCTTCGAAGATCTGACCCGCTCCACAAC	pMSP12:: Wasabi	pSS P_{msp12}DevR-Myc and pSS P_{msp12}DevR$_{\Delta\alpha10}$
msp12 NdeI R	GCCGCCCATATGTATATCTCCTTCTTAAATC		
rrn pro Bst BI F	GCGGCCTTCGAATCGTGGAGAACCTGGTGAGTCTC	Mtb H37Rv DNA	pSS P_{rrn}DevR-Myc
rrn-gfprbs-Ndel R	CCGCATATGTATAT**CTCCTTC**TTACGCCGCCAGCGTTCGTCCTG		
devR D54E F	GCGGTGCTGGAGGTCCGGTTGCCCGAT	pSS P_{msp12}DevR-Myc	pSS P_{msp12}DevR D54E-Myc
devR D54E R	CAACCGGACCTCCAGCACCGCGACATC		
devR T82AF	TGTCTGATCCTCGCGTCCTACACCTCT	pSS P_{msp12}DevR-Myc	pSS P_{msp12}DevR T82A-Myc
devR T82AR	AGAGGTGTAGGACGCGAGGATCAGACA		

Cloning sites are underlined.
Sequence highlighted in bold is RBS (ribosome binding site).
