# Supplementary material for: Mycobacterium tuberculosis DevR/DosR Dormancy Regulator Activation Mechanism: Dispensability of Phosphorylation, Cooperativity and Essentiality of α10 Helix
Source: PLoS One. 2016 Aug 4;11(8):e0160723. doi: 10.1371/journal.pone.0160723 (PMC4973870; doi:10.1371/journal.pone.0160723)
Supplement: S3 Table — (PDF) [file pone.0160723.s003.pdf]

S3 Table. Oligonucleotide primers used for qPCR

Gene (Rv no.)	Primer Name	Sequence 5' → 3'
Rv0571c	*Rv0571c* F	ACG GTG CGG ACA AGG TGG TGC TG
	Rv0571c R	CGC CAA ACA CAC CAC CTC ATC GG
narK2 (1737c)	*narK2* F	CTG GTA CCA GCC GGC GCG
	narK2 R	AAC CGC GGG GTG AAG AAC GC
Rv1738	*Rv1738* F	CGA CGA ACA CGA AGG ATT GA
	Rv1738 R	ACA CCC ACC AAT TCC TTT TCC
ctpF (Rv1997)	*ctpF* F	CAG CAC CAC GGT CAT CTG
	ctpF R	ATC TCA CCG TGG GGT GTC
otsB1 (Rv2006)	*otsB1* F	GAT CTA CCC TGA GCG CTG TC
	otsB1 R	GAT ATC GGA GCG CAG GAC
fdxA (Rv2007c)	*fdxA* F	TGT CCG GTC GAC TGT ATC TAT GA
	fdxA R	GGC AGG CCG GTT TGC
hspX (Rv2031c)	*hspX* F	CGC ACC GAG CAG AAG GA
	hspX R	ACC GTG CGA ACG AAG GAA
acg (Rv2032)	*acg* F	GCT TTT GAG ACT TCT GAG GGC ATA
	acg R	GGT GAC CCG GTC ACT TTC G
tgs1 (Rv3130c)	*tgs1* F	TGG CTG CCG GGC CTT TCC C
	tgs1 R	GCA GGG CCA AAG GTC CTC C
Rv3131	*Rv3131* F	CGA TCA GGC CGA TGT CGC CTT
	Rv3131 R	TCA CCT CCT GGC ACC GGC C
devR (Rv3133c)	*devR* F	CCG ATC TGC GCT GTC TGA TC
	devR R	GTC CAG CGC CCA CAT CTT T
Rv3134c	*Rv3134c* F	CTG GCT GGG TCG GCC TTA
	Rv3134c R	GCT GAC CTG GGA GGT TGT CG
